# Radioanatomical relationships of the carotid arteries to the superior cornu of the thyroid cartilage

**DOI:** 10.1007/s00276-026-03915-w

**Published:** 2026-06-09

**Authors:** Nektaria Karangeli, George Triantafyllou, Panagiotis Papadopoulos-Manolarakis, Nikolaos-Achilleas Arkoudis, Georgios Velonakis, Panagiotis Papanagiotou, Maria Piagkou

**Affiliations:** 1 Department of Anatomy, Faculty of Health Sciences, School of Medicine, National and Kapodistrian University of Athens, 75 Mikras Asias str., Goudi, 11527 Athens, Greece; 2 Department of Neurosurgery, General Hospital of Nikaia-Piraeus, Athens, Greece; 3 Research Unit of Radiology and Medical Imaging, National and Kapodistrian University of Athens, Athens, Greece; 4 Second Department of Radiology, General University Hospital “Attikon”, National and Kapodistrian University of Athens, Athens, Greece; 5 First Department of Radiology, “Aretaieion” Hospital, National and Kapodistrian University of Athens, Athens, Greece

**Keywords:** Carotid arteries, Thyroid cartilage, Superior cornu, Computed tomography angiography, Anatomical variation

## Abstract

**Purposes:**

The carotid arteries demonstrate variable relationships with adjacent laryngeal structures. Although thyroid cartilage (TC) is commonly used as a surgical landmark, the relationship between the carotid arteries and the superior cornu of the thyroid cartilage (SCTC) remains insufficiently quantified. Previous clinical reports have suggested that close proximity between the vessel and cartilage may have anatomical significance in selected cases of vascular compression. This study aimed to evaluate these relationships and propose a classification system.

**Methods:**

A retrospective analysis of 214 head and neck computed tomography angiography (CTA) scans (153 males, 61 females; mean age 63.07 years) was performed. The level of the carotid bifurcation (CB) relative to the SCTC, spatial relationships, and minimum distances between vessels and the SCTC were recorded. SCTC morphology, length, and angulation were also assessed.

**Results:**

The CB was suprathyroid in 89% of sides and infrathyroid in 11%. The CCA was lateral to the SCTC in 73.4% of sides, whereas the ECA and ICA were lateral in 19.6% and 11%, respectively. Medial configurations were uncommon. Mean distances increased from the CCA (2.33 ± 1.82 mm) to the ECA (4.38 ± 3.07 mm) and ICA (6.18 ± 3.67 mm). SCTC length demonstrated a moderate positive correlation with ECA distance (R = 0.401), while coronal angulation showed weak negative correlations with ECA and ICA distances. Eleven relationship types were identified, with Type 1 (CCA lateral) representing the predominant configuration (64.5%).

**Conclusions:**

A consistent proximity pattern was observed, with the CCA located closest to the SCTC, followed by the ECA and ICA. SCTC morphology modestly contributed to carotid artery positioning. The proposed classification system captures the variability of these anatomical relationships and may facilitate standardized radiological description and preoperative anatomical assessment. Further studies incorporating dynamic imaging and clinical correlation are required to determine the potential pathological significance of these configurations.

## Introduction

The level and orientation of the common carotid artery bifurcation (CB) exhibit considerable interindividual variability. Despite this variation, the superior cornu of the thyroid cartilage (SCTC) is commonly used as a surface landmark to approximate the CB [7, 8, 13]. Consequently, it serves as a practical anatomical reference during clinical examination and surgical procedures involving the cervical region.

The common carotid artery (CCA) usually bifurcates into the internal (ICA) and external (ECA) carotid arteries at the level of the superior border of the thyroid cartilage (TC) [13]. However, the vertical level of the CB is highly variable, with reports describing locations ranging from the level of the hyoid bone (HB) to the cricoid cartilage [8]. Furthermore, while the ICA typically maintains a posterolateral position relative to the ECA at the origin, their courses relative to the laryngeal framework can shift significantly due to vascular tortuosity or anatomical constraints [10].

During their cervical course, the carotid arteries demonstrate variable relationships with adjacent osteocartilaginous and soft-tissue structures, including the TC, HB [9], styloid process [16], mandible [2], and cervical vertebrae [13]. These relationships may influence local hemodynamics and are particularly relevant during surgical and interventional procedures in the parapharyngeal and suprahyoid regions [18]. Unlike the HB, the SCTC is a relatively inferior and stable laryngeal landmark that lies in close proximity to the CB and proximal carotid arteries in a substantial proportion of individuals.

Recent clinical reports have highlighted the potential anatomical significance of these relationships by describing cases of carotid artery impingement against the SCTC associated with thromboembolic events and ischemic stroke [3, 5, 15]. Dynamic imaging studies have further demonstrated that swallowing and cervical motion may accentuate vessel-cartilage contact in selected individuals [19]. Nevertheless, despite increasing recognition of carotid compression syndromes involving laryngeal structures, the spatial relationship between the carotid arteries and the SCTC remains insufficiently characterized, with most available evidence limited to isolated anatomical observations or case reports [3, 12, 15].

Therefore, the present study aimed to provide a quantitative computed tomography angiography-based assessment of the relationship between the carotid arteries and the SCTC, including spatial orientation, morphometric measurements, and anatomical variability. In addition, a classification system was developed to facilitate standardized radiological and anatomical descriptions of these configurations.

## Materials and methods

### Sample

This retrospective study included 214 head and neck CTA scans acquired at the General Hospital of Nikaia–Piraeus during routine clinical practice. Ethical approval was obtained from the institutional review board (approval number: 56485/13.11.2024). The scans were performed between January 2020 and December 2023. The cohort consisted of 153 males and 61 females, with a mean age of 63.07 ± 13.8 years (range: 20–89 years).

### Radiological protocols

All examinations were performed using a 128-slice CT scanner (SOMATOM go. Top, Siemens Healthineers). Patients were positioned supine with the head in neutral alignment. An iodine-based contrast agent (60 mL) was administered intravenously at a rate of 4.0–4.5 mL/s. Image acquisition was performed with a slice thickness of 0.6–0.8 mm. Datasets with inadequate image quality or anatomical distortion due to pathology (e.g., tumors, trauma, prior cervical surgery, extensive calcification, or congenital anomalies) were excluded. Mild age-related changes, including non-stenotic atherosclerosis and minor vascular elongations, were not grounds for exclusion, as these represent common findings in the general population and were not considered sufficient to substantially alter the topographic relationships under investigation.

Image analysis was conducted using Horos software (Horos Project). Multiplanar reconstructions (axial, coronal, sagittal) and three-dimensional volume-rendered images were used for anatomical assessment. All measurements were independently performed by two observers experienced in head and neck anatomical imaging analysis.

### Morphological assessment and measurements

**Fig. 1:**
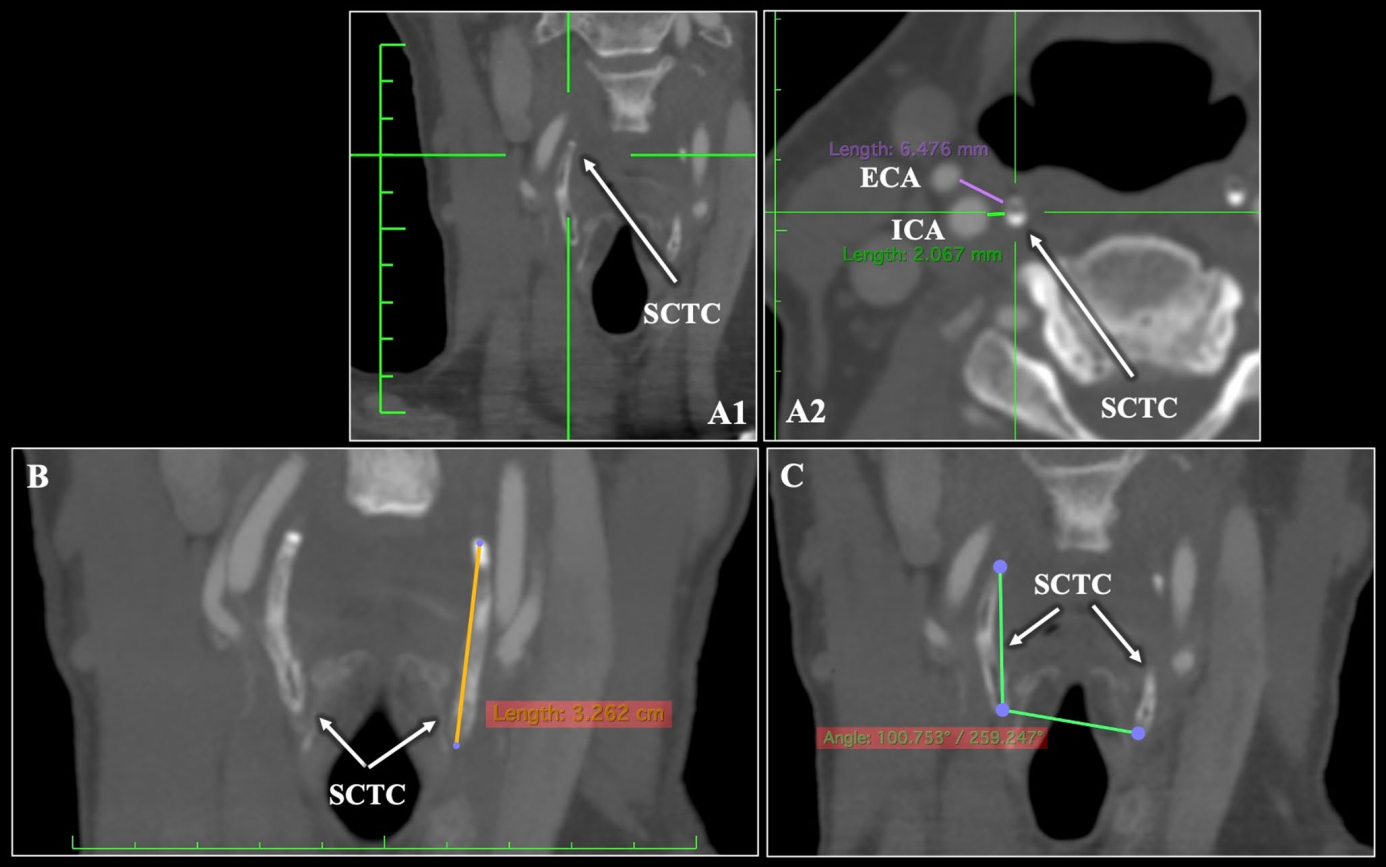
Measurement methodology. **A1** Identification of the superior cornu of the thyroid cartilage (SCTC) on coronal reconstruction. **A2** Measurement of the minimum linear distance between the carotid arteries and the SCTC on axial reconstruction. **B** Measurement of SCTC length on coronal reconstruction. **C** Measurement of SCTC angulation. ECA, external carotid artery; ICA, internal carotid artery

The level of the CB was determined on sagittal reconstructions and classified as suprathyroid or infrathyroid relative to the SCTC. The spatial relationships of the CCA, ICA, and ECA to the SCTC were evaluated on axial and three-dimensional reconstructions and categorized as lateral, medial, or absent. Distance measurements were performed on axial images at the point of closest approximation between the SCTC and the respective vessels, and the shortest linear distance between their outer boundaries was recorded (Fig. 1). Measurements were obtained using standardized multiplanar reconstructions in order to minimize orientation-related variability.

In suprathyroid CB configurations, the distance between the CCA and the SCTC was measured. In infrathyroid configurations, the distances between the SCTC and the ICA and ECA were measured independently. When borderline configurations were encountered, classification was determined based on the predominant vessel trajectory relative to the SCTC, following consensus review between observers.

SCTC morphology was assessed by recording anatomical variations and the presence of overlap with the HB, in accordance with established anatomical descriptions [11]. SCTC morphometry included measurement of length on coronal reconstructions and angulation on both coronal and sagittal planes (Fig. 1).

### Classification system

Based on the observed configurations, a classification system comprising three groups and eleven types was developed. Type 0 indicated no relationship between the SCTC and the carotid arteries. Group A variants had a single vessel in relationship with the SCTC, and Types 1–5 belonged to this group. Type 1 corresponded to the CCA positioned laterally to the SCTC, whereas Type 2 described a medial CCA. Type 3 and Type 4 represented lateral positioning of the ECA and ICA, respectively, while Type 5 corresponded to a medial ICA. Group B variants had two vessels in proximity with the SCTC, and Types 6–9 belonged to this group. Type 6 included both ECA and ICA positioned laterally, and Type 7 described a lateral ECA with a medial ICA. Type 8 and Type 9 represented lateral positions of the CCA relative to the ECA and ICA, respectively. Group C had all three vessels in relationship with the SCTC, and only Type 10 belonged to this group. Type 10 described all three arteries (CCA, ICA, and ECA) positioned laterally to the SCTC.

### Statistical analysis

Statistical analysis was performed using IBM SPSS Statistics (version 30, IBM Corp., Armonk, NY, USA). Categorical variables were compared using the Chi-square test for unpaired data and McNemar’s test for paired data. Normality was assessed with the Shapiro–Wilk test. Continuous variables were analyzed using the independent t-test or Mann–Whitney U test for unpaired data, and the paired t-test for paired data. Comparisons among more than two groups were performed using one-way ANOVA or the Kruskal–Wallis test, as appropriate. Correlation analysis was conducted using Pearson or Spearman coefficients, depending on data distribution. Results are presented as mean ± standard deviation. Correlation strength was interpreted as weak (R < 0.30), moderate (R = 0.30–0.49), or strong (R ≥ 0.50). To minimize the risk of Type I error due to multiple comparisons, a family-wise Bonferroni correction was applied. Statistical significance for differences between vessels, sex, and sides (11 tests) was set at *p* < 0.0045, for morphometric dimorphism (7 tests) at *p* < 0.0071, and for correlation analyses (9 tests) at *p* < 0.0055. Results that did not meet the adjusted significance thresholds were interpreted as nominally significant.

All datasets were evaluated independently by two observers (NK, GT). In cases of disagreement, consensus was reached after review by the senior authors (PPM, NAA). Interobserver reliability for categorical variables was assessed using Cohen’s kappa (κ), with values ≥ 0.60 indicating acceptable agreement. For continuous variables, reliability was evaluated using the intraclass correlation coefficient (ICC), with values ≥ 0.75 indicating good agreement.

## Results

Interobserver agreement was excellent, with Cohen’s kappa for categorical variables being 0.931, indicating almost perfect agreement. The ICC for morphometric measurements was 0.89, demonstrating high reliability.

### Carotid artery variations

**Fig. 2:**
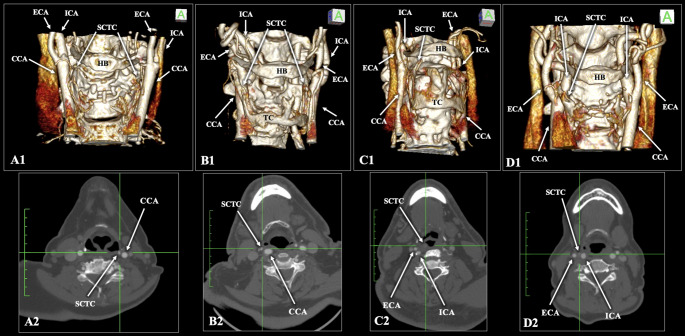
Representative examples of carotid arteries with lateral or medial trajectories compared to the superior cornu of the thyroid cartilage (SCTC). Three-dimensional (**A1**, **B1**, **C1**, **D1**) and axial (**A2**, **B2**, **C2**, **D2**) reconstructions are depicted. Patient (A) exhibits the lateral common carotid artery (CCA). Patient (B) has medial CCA. Patient (C) depicts lateral internal and external carotid artery (ICA and ECA). Patient (D) exhibits medial ICA

The CB was located above the SCTC in 381 sides (89%) and below in 47 sides (11%). The CB showed no significant association with sex (*p* = 0.055) or side (*p* = 0.877) (Table 1). The CCA was located lateral to the SCTC in 314 sides (73.4%) and medial in 5 sides (1.2%), whereas no relationship was observed in 62 sides (14.7%) (Fig. 2). Sex showed a significant association with the CCA–SCTC relationship (*p* < 0.001), whereas side and age were not significant (*p* = 0.942 and *p* = 0.230, respectively) (Table 1).

**Table 1:** Distribution of carotid bifurcation (CB) level and arterial relationships with the superior cornu of the thyroid cartilage (SCTC). Bold p-values with asterisk (*) signifies statistically significant results

	Total N = 428 (frequency-%)	Left n = 214	Right n = 214	*p*-value	Females n = 122	Males n = 306	*p*-value
*CB*							
Above SCTC	381 (89)	190	191	0.877	103	278	0.055
Below SCTC	47 (11)	24	23		19	28	
*SCTC-CCA relationship*							
Lateral	314 (73.4)	155	159	0.942	71	243	< 0.001*
Medial	5 (1.2)	3	2		3	2	
No relationship	62 (14.7)	32	30		29	33	
*SCTC-ECA relationship*							
Lateral	84 (19.6)	38	46	0.519	29	55	0.129
Medial	0 (0)	0	0		0	0	
No relationship	19 (4.4)	11	8		8	11	
*SCTC-ICA relationship*							
Lateral	47 (11)	22	25	0.142	15	32	0.003*
Medial	5 (1.2)	0	5		5	0	
No relationship	51 (11.9)	27	24		17	34	

Data are presented as the number of sides (%). SCTC: superior cornu of the thyroid cartilage; CCA: common carotid artery; ECA: external carotid artery; ICA: internal carotid artery

The ECA was located lateral to the SCTC in 84 sides (19.6%), while no relationship was identified in 19 sides (4.4%) (Fig. 2). No significant associations were found for sex (*p* = 0.129), side (*p* = 0.519), or age (*p* = 0.767) (Table 1).

The ICA was located lateral to the SCTC in 47 sides (11%) and medial in 5 sides (1.2%), while no relationship was observed in 51 sides (11.9%) (Fig. 2). Sex showed a significant association with the ICA–SCTC relationship (*p* = 0.003), whereas side and age were not significant (*p* = 0.142 and *p* = 0.074, respectively) (Table 1).

**Fig. 3:**
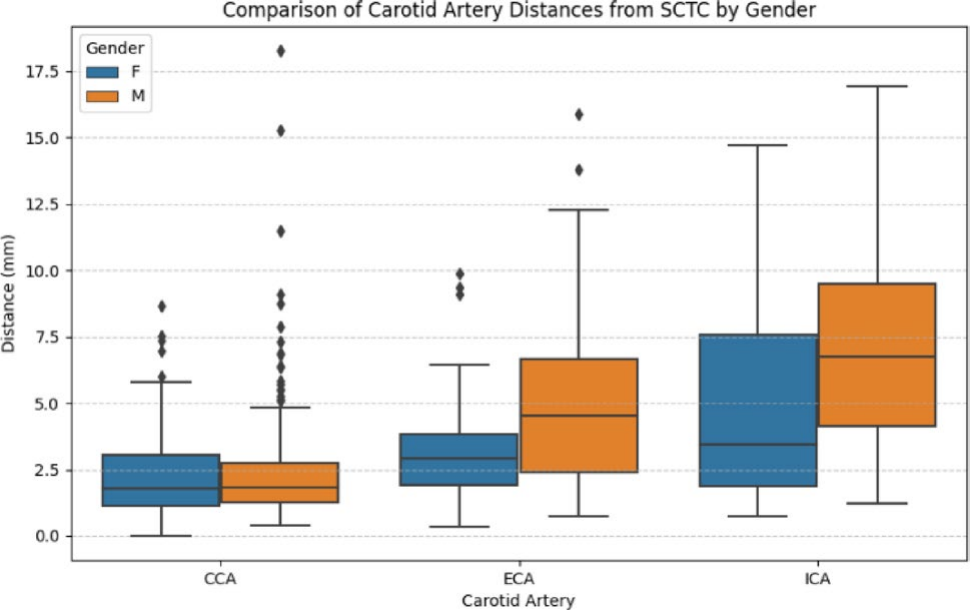
Boxplots for the sexual dimorphism of the distances between the carotid arteries and the superior cornu of the thyroid cartilage (SCTC). CCA- common carotid artery, ICA- internal carotid artery, ECA- external carotid artery

The mean distances were 2.33 ± 1.82 mm for the CCA–SCTC, 4.38 ± 3.07 mm for the ECA–SCTC, and 6.18 ± 3.67 mm for the ICA–SCTC. Significant sex differences were observed for the ICA–SCTC distance (*p* = 0.001) (Fig. 3, Table 2). Although nominal sex differences were observed for the ECA–SCTC distance (*p* = 0.018), this finding did not remain statistically significant following Bonferroni correction.

**Table 2:** Descriptive statistics of the morphometric parameters of the current study

Distances	Total N = 428	Left n = 214	Right n = 214	*p*-value	Females n = 122	Males n = 306	*p*-value
SCTC-CCA	2.33 ± 1.82	2.33 ± 2.03	2.32 ± 1.59	0.565	2.30 ± 1.57	2.33 ± 1.91	0.961
SCTC-ECA	4.38 ± 3.07	4.24 ± 3.10	4.50 ± 3.07	0.499	3.33 ± 2.26	4.96 ± 3.32	0.018
SCTC-ICA	6.18 ± 3.67	6.74 ± 3.97	5.66 ± 3.32	0.238	4.75 ± 3.54	6.99 ± 3.52	0.001*

Values are presented as mean ± standard deviation. SCTC: superior cornu of the thyroid cartilage; CCA: common carotid artery; ECA: external carotid artery; ICA: internal carotid artery

Values are presented as mean (standard deviation) in mm

### Superior cornu of the thyroid cartilage

Typical SCTC anatomy was observed in 306 sides (71.5%), whereas variants were identified in 122 sides (28.5%). SCTC morphology was not associated with sex (*p* = 0.182) or side (*p* = 0.979) (Table 3). Overlap between the SCTC and the HB was observed in 20 sides (4.7%), with no significant association with sex (*p* = 0.171) or side (*p* = 0.360) (Table 4).

**Table 3:** Descriptive statistics of the morphological parameters of the current study

SCTC variations	Total N = 428 (frequency %)	Left (n = 214)	Right (n = 214)	*p*-value	Females (n = 122)	Males (n = 306)	*p*-value
Typical morphology	306 (71.5%)	155	151	0.979	82	224	0.182
Triticeal cartilage	116 (27.1%)	56	60		40	76	
Articulation with triticeal Cartilage	4 (0.9%)	2	2		0	4	
Articulation with GHHB	2 (0.5%)	1	1		0	2	

All the values are presented as Cases (Frequency), GHHB-greater horn of the hyoid bone

**Table 4:** Overlap between the superior cornu of the thyroid cartilage (SCTC) and the hyoid bone (HB), N = total number of cases

Overlap with HB	Total N = 428 (frequency %)	Left n = 214	Right n = 214	*p*-value	Females n = 122	Males n = 306	*p*-value
No	408 (95.3%)	208	202	0.360	119	289	0.171
Yes	20 (4.7%)	8	12		3	17	

**Fig. 4:**
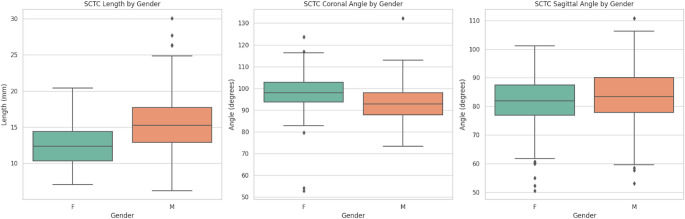
Boxplots for the sexual dimorphism of the measurements of the superior cornu of the thyroid cartilage (SCTC)

The mean SCTC length was 14.66 ± 4.04 mm, with a coronal angle of 94.19 ± 8.36° and a sagittal angle of 82.76 ± 9.26°. SCTC length and coronal angulation demonstrated significant sexual dimorphism (*p* < 0.001), whereas sagittal angulation showed only nominal significance (*p* = 0.039) after Bonferroni adjustment (Fig. 4, Table 5).

**Table 5:** Morphometric measurements of the superior cornu of the thyroid cartilage (SCTC)

Parameters	Total N = 428 (frequency %)	Left n = 214	Right n = 214	*p*-value	Females n = 122	Males n = 306	*p*-value
SCTC length	14.66 (4.04)	14.36 (3.90)	14.95 (4.17)	0.246	12.59 (3.04)	15.48 (4.10)	< 0.001*
SCTC coronal angulation	94.19 (8.35)	92.78 (8.21)	95.60 (8.28)	< 0.001*	97.79 (9.06)	92.76 (7.61)	< 0.001*
SCTC sagittal angulation	82.76 (9.26)	80.41 (9.71)	85.10 (8.17)	< 0.001*	80.90 (9.69)	83.50 (8.99)	0.039

### Comparative and correlation analysis

The distance between the CCA and the SCTC differed significantly by spatial relationship (*p* < 0.001), with medial positioning showing the shortest distance. The ECA–SCTC distance did not differ significantly between relationship types (*p* = 0.101). The ICA–SCTC distance varied significantly (*p* < 0.001), with medial positioning associated with shorter distances.

No significant associations were observed between SCTC morphological variants and arterial distances (CCA: *p* = 0.752; ECA: *p* = 0.342; ICA: *p* = 0.094). Similarly, overlap with HB was not associated with arterial distances (CCA: *p* = 0.525; ECA: p = 0.316; ICA: *p* = 0.770).

A positive moderate correlation was identified between SCTC length and ECA–SCTC distance (R = 0.401, *p* < 0.001). Negative weak correlations were observed between SCTC coronal angulation and both ECA–SCTC distance (R =  − 0.227, *p* = 0.023) and ICA–SCTC distance (R =  − 0.266, *p* = 0.008) (Table 6).

**Table 6:** Correlation between SCTC morphometry and carotid artery distances

SCTC parameters	Arterial distance from the SCTC	Correlation coefficient (R)	*p*-value
Length	CCA-SCTC	− 0.018	0.705
	ECA-SCTC	+ 0.401	< 0.001*
	ICA-SCTC	0.099	0.329
Coronal angulation	CCA-SCTC	− 0.017	0.721
	ECA-SCTC	− 0.227	0.023
	ICA-SCTC	− 0.266	0.008
Sagittal angulation	CCA-SCTC	− 0.027	0.575
	ECA-SCTC	− 0.066	0.512
	ICA-SCTC	0.089	0.378

### Classification system

Type 0 (no arterial relationship) was identified in 50 sides (11.7%).

**Fig. 5:**
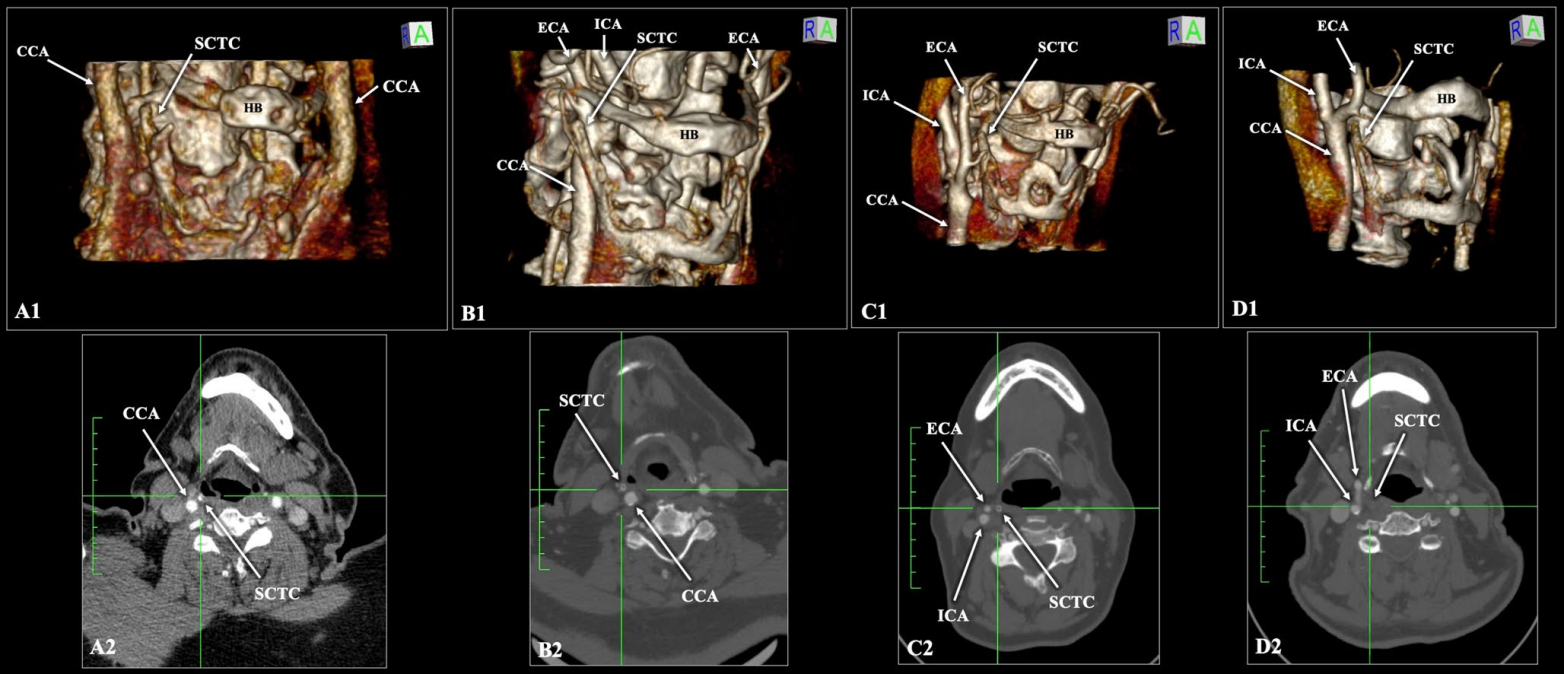
The proposed classification system (Type 1–4) depicted through three-dimensional (**A1**, **B1**, **C1**, **D1**) and axial (**A2**, **B2**, **C2**, **D2**) reconstructions. Patient (A) has Type 1 with the common carotid artery (CCA) lateral. Patient (B) has Type 2 with CCA medial. Patient (C) has Type 3 with external carotid artery (ECA) lateral. Patient (D) has Type 4 with internal carotid artery (ICA) lateral

Among single-artery relationships (Group A, Types 1–5), Type 1 (CCA lateral) was the most frequent, observed in 276 sides (64.5%) (Fig. 5). Type 2 (CCA medial) occurred in 5 sides (1.2%), Type 3 (ECA lateral) in 32 sides (7.5%), Type 4 (ICA lateral) in 4 sides (0.9%), and Type 5 (ICA medial) in 1 side (0.2%).

**Fig. 6:**
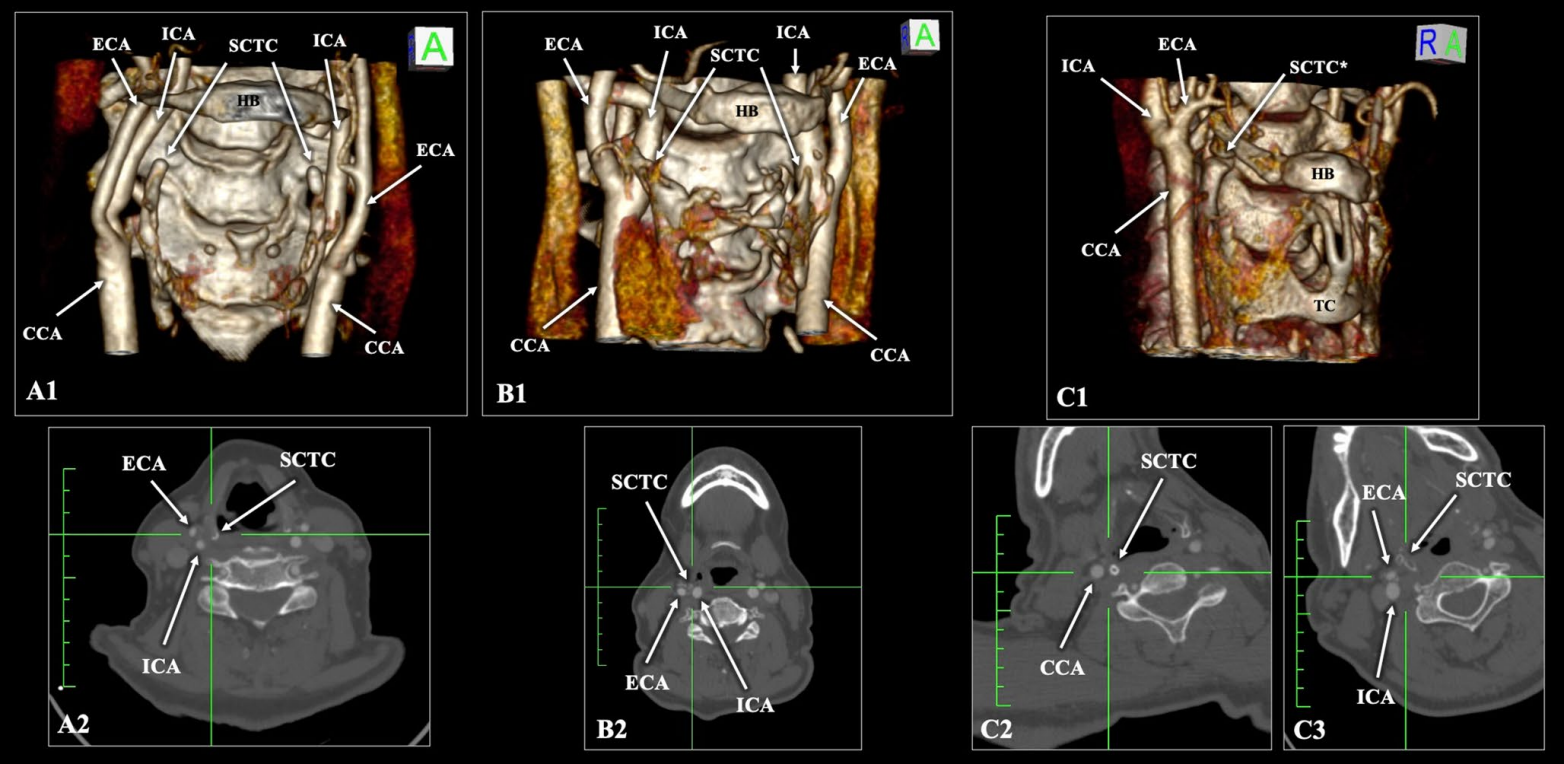
The proposed classification system (Type 6–8) is depicted through three-dimensional (**A1**, **B1**, **C1**) and axial (**A2**, **B2**, **C2**) reconstructions. Patient (A) has Type 6 with the internal and external carotid artery (ICA and ECA) lateral. Patient (B) has Type 7 with ICA medial and ECA lateral. Patient (C) has Type 8 with the common carotid artery (CCA) lateral and the ECA lateral

**Fig. 7:**
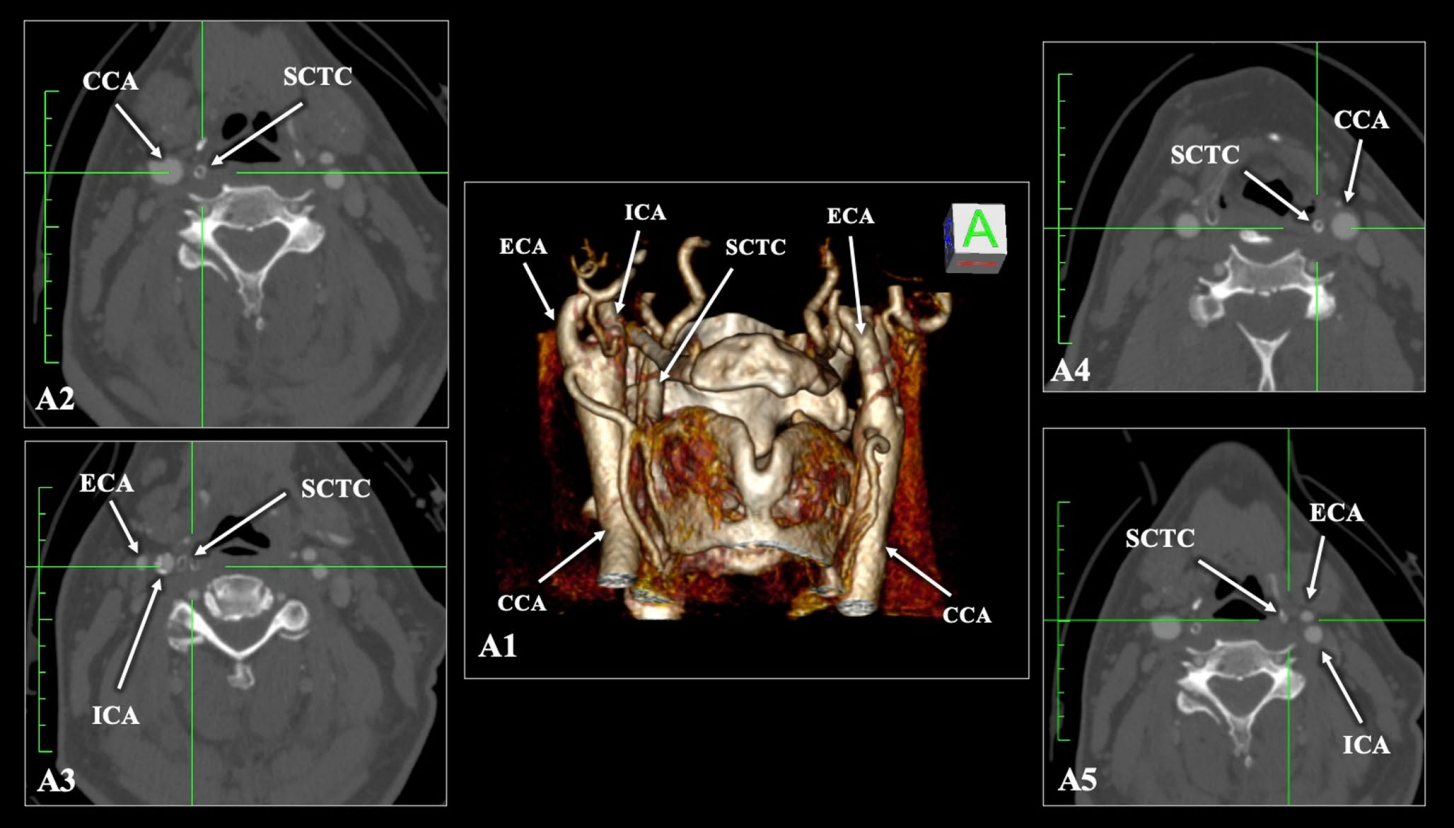
The proposed classification system (Type 9–10) is depicted through three-dimensional (**A1**) and axial (**A2**–**A5**) reconstructions. Patient (A) has Type 9 on the right side with common carotid artery (CCA) and internal carotid artery (ICA) lateral, while on the left side has Type 10 with CCA, ICA, and external carotid artery (ECA) lateral

Among dual-artery relationships (Group B, Types 6–9), Type 6 (ECA and ICA lateral) was observed in 18 sides (4.2%), Type 7 (ECA lateral and ICA medial) in 4 sides (0.9%), Type 8 (CCA and ECA lateral) in 13 sides (3%), and Type 9 (CCA and ICA lateral) in 8 sides (1.9%) (Figs. 6–7).

Type 10 (all arteries lateral) was identified in 17 sides (4%) (Fig. 7).

Bilateral symmetry was observed in 134 patients, whereas 80 patients showed asymmetrical configurations. Sex differences were identified in classification distribution (*p* < 0.001) (Table 7). Arterial distances differed significantly across classification types (*p* < 0.001).

**Table 7:** Distribution of the proposed classification system

Classification into types	Total N = 428 (frequency %)	Left n = 214	Right n = 214	*p*-value	Females n = 122	Males n = 306	*p*-value
0	50 (11.7)	27	23	0.582	21	29	< 0.001*
1	276 (64.5)	139	137		63	213	
2	5 (1.2)	3	2		3	2	
3	32 (7.5)	18	14		12	20	
4	4 (0.9)	3	1		2	2	
5	1 (0.2)	0	1		1	0	
6	18 (4.2)	8	10		8	10	
7	4 (0.9)	0	4		4	0	
8	13 (3)	5	8		3	10	
9	8 (1.9)	4	4		3	5	
10	17 (4)	7	10		2	15	

## Discussion

**Fig. 8:**
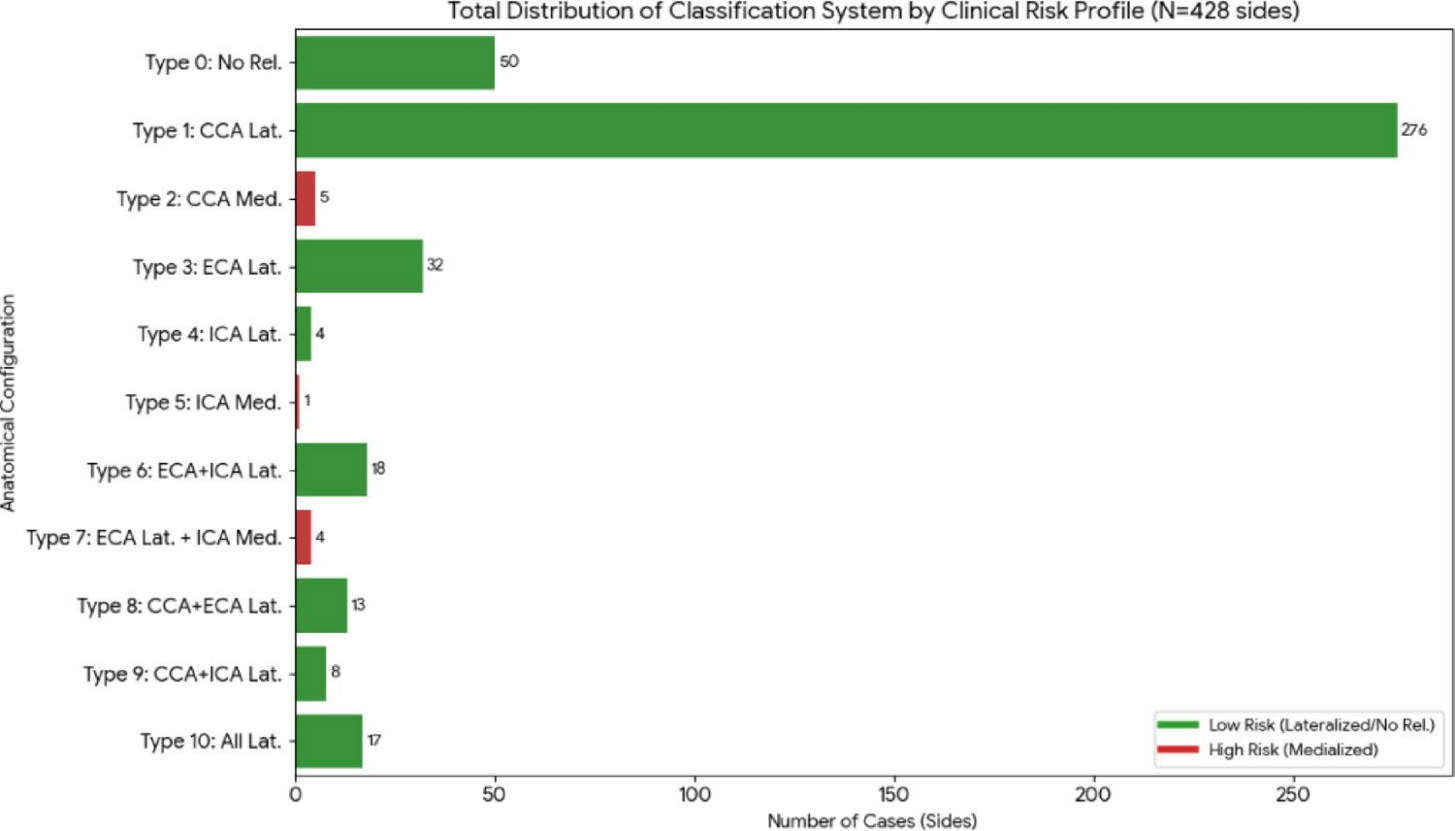
Frequency distribution of the proposed classification types. CCA- common carotid artery, ICA- internal carotid artery, ECA- external carotid artery

This study characterizes the anatomical relationship between the carotid arteries and the SCTC, demonstrating considerable variability. A consistent proximity pattern was observed, with the CCA closest to the SCTC, followed by the ECA and ICA. The proposed 11-type classification system enabled the identification of less common configurations, particularly Types 2, 5, and 7, in which the arteries course medially to the SCTC and, in theory, could facilitate closer vessel–cartilage proximity (Fig. 8).

**Fig. 9:**
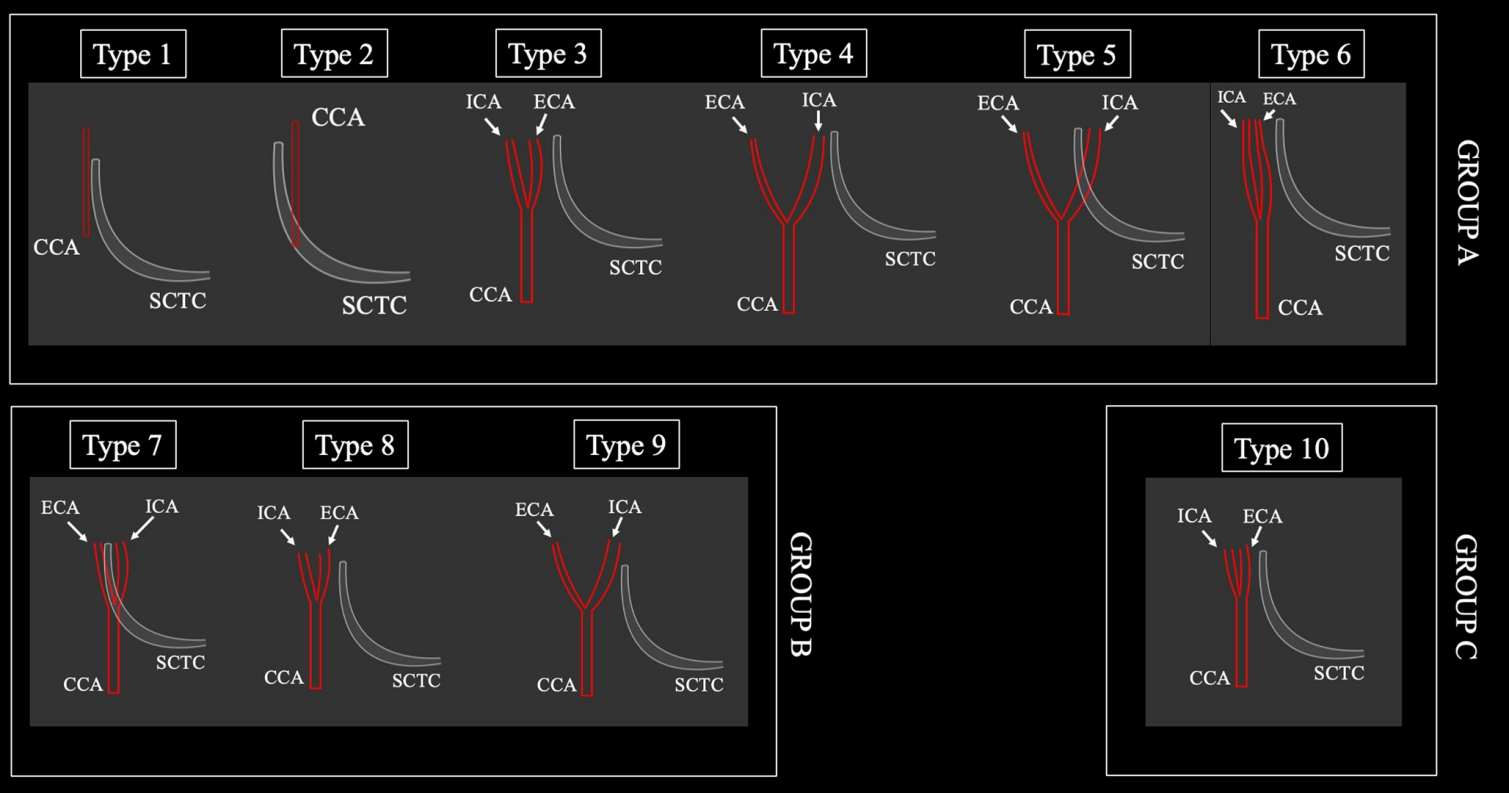
Schematic representation of the proposed classification system of carotid artery–superior cornu of the thyroid cartilage (SCTC) relationships. Detailed group (Group A-C) and btype classification (Types 0–10) according to the topographic relationship of the common carotid artery (CCA), internal carotid artery (ICA), and external carotid artery (ECA) to the SCTC

Although eleven configurations were identified, the majority of cases were represented by a limited number of dominant patterns, particularly Type 1. Therefore, the classification should not be interpreted as implying equal anatomical or clinical significance across all categories. Rather, it was developed as a descriptive anatomical framework to systematically document both common and uncommon carotid artery–SCTC relationships. From a practical perspective, the observed configurations may also be conceptually grouped into absent, lateral, medial, and combined relationship patterns while retaining the detailed subtype structure for anatomical and radiological documentation (Fig. 9).

Sex-related differences were observed in SCTC dimensions and arterial distances, suggesting that laryngeal morphology contributes to regional vascular arrangement. Nevertheless, the dimorphism observed in this study likely reflects a combination of size scaling and true morphological differences. While the greater linear distances in males may be partially attributed to overall larger cervical dimensions, the significant differences in coronal angulation indicate that the three-dimensional geometry of the thyroid cartilage differs between sexes. Although sagittal angulation demonstrated nominal significance, this finding did not remain significant following Bonferroni correction and should therefore be interpreted cautiously. In addition, correlations between SCTC angulation and arterial proximity suggest that cartilage geometry may contribute to vessel position and relative proximity, although the observed associations were generally weak-to-moderate in magnitude.

SCTC morphometry showed specific associations with vascular distances. Increased SCTC length was associated with greater ECA–SCTC distance, whereas increased coronal angulation was associated with reduced distances to both the ECA and ICA. These findings suggest that variation in the three-dimensional configuration of the thyroid cartilage contributes to the position of adjacent vessels. However, the observed correlations were generally weak-to-moderate in magnitude, indicating that SCTC morphology likely represents only one component of a multifactorial anatomical relationship. Although such relationships have not been systematically quantified, previous morphometric studies provide indirect support for the influence of laryngeal skeletal morphology on adjacent cervical vascular topography [12].

The classification system proposed in this study builds on existing landmark-based approaches, such as the HB–centered model by Manta et al. (2024). It defines eleven configurations based on the position of the carotid arteries relative to the SCTC, incorporating both single- and multi-vessel relationships as well as medial and lateral orientations. Unlike the HB, the SCTC is a relatively stable laryngeal landmark located inferior to the hyoid bone that lies in close proximity to the carotid bifurcation and proximal carotid arteries in a substantial proportion of individuals. This framework allows standardized description and facilitates correlation with imaging and surgical anatomy. Although several subtypes were uncommon, they were retained in order to comprehensively document the full spectrum of observed anatomical variability. From a practical perspective, the classification may additionally be simplified into broader absent, lateral, and medial, and combined relationship patterns for easier clinical interpretation.

Clinical reports indicate that contact between cervical arteries and adjacent osteocartilaginous structures may lead to vascular compression and ischemic events [1, 3, 5, 6, 14, 15, 17, 19]. Repetitive impingement of the CCA by the TC has been associated with endothelial injury, thrombus formation, and stroke [3, 6, 15]. Dynamic imaging studies have further shown that positional changes, such as neck rotation and swallowing, can accentuate arterial compression, particularly in cases of medial displacement [19]. Karle et al. (2019) also described carotid compression by the SCTC associated with stroke, underscoring the clinical relevance of these relationships [5]. Nevertheless, the present study was not designed to establish a direct causal association between the observed anatomical configurations and cerebrovascular events.

Surgical observations support these findings, as the TC or HB may limit exposure during carotid endarterectomy, occasionally requiring partial resection [4]. While our study does not assess hemodynamics, the proximity observed in certain variants suggests a potential anatomical substrate for mechanical interaction, as described in previous clinical reports of carotid compression [3, 5, 15, 17, 19]. Accordingly, the present findings should primarily be interpreted as demonstrating anatomical proximity patterns rather than direct evidence of pathological compression.

In this context, the present classification highlights configurations that could, in principle, facilitate closer vessel–cartilage approximation. Medial patterns (Types 2, 5, and 7) may create anatomical conditions favoring arterial contact with the SCTC, particularly during cervical movement or swallowing. Although these variants were relatively infrequent, their anatomical proximity patterns may warrant further investigation. In contrast, lateral configurations, which were considerably more common, are less likely to result in direct vessel–cartilage approximation but remain relevant for radiological interpretation and surgical planning.

This study has several limitations. First, measurements were obtained from CTA scans acquired in the supine position with the head in neutral alignment; therefore, dynamic changes during movement were not assessed. Second, CTA does not fully depict soft tissue structures, such as fascia and ligaments, that may influence vessel mobility. Thus, while we identified clear correlations between SCTC dimensions and vascular distances, we must acknowledge that our study does not account for all potential anatomical confounders (such as fasciae and ligaments, neck length, the degree of cervical spine lordosis, and the thickness of the parapharyngeal fat pad). Third, the single-center design may limit generalizability. Finally, as this was a retrospective radioanatomical study, we were unable to correlate the observed anatomical variants with the patients’ clinical histories or long-term vascular outcomes. In addition, the low frequency of certain subtypes limits statistical power for subgroup-specific interpretation. Future studies incorporating dynamic imaging and prospective clinical correlation are required to clarify whether these anatomical configurations contribute to symptomatic vascular compression or cerebrovascular events.

## Conclusions

This study provides a radiological assessment of the relationship between the carotid arteries and the SCTC. A consistent proximity pattern was observed, with the CCA closest to the SCTC, followed by the ECA and ICA. This relationship was modestly influenced by SCTC length and coronal angulation. The proposed eleven-type classification system captures the variability of these relationships and distinguishes common lateral configurations from less frequent medial variants. Although the majority of cases were characterized by dominant lateral patterns, the classification also enabled documentation of uncommon medial configurations that may indicate closer vessel–cartilage proximity. Medial patterns warrant further investigation, as their anatomical relationship to the SCTC could theoretically facilitate vessel–cartilage contact during dynamic neck movements. Sex-related differences in SCTC morphology and arterial distances further support the importance of individualized anatomical assessment in imaging interpretation and surgical planning. Future studies incorporating dynamic imaging and clinical correlation are necessary to determine the potential pathological significance of these anatomical relationships.

## Data Availability

All the data are available upon reasonable request to the corresponding author.
